# Supplementary material for: Context-dependent siderophore exploitability shapes microbial community structure
Source: ISME J. 2026 Sep 16;20(1):wrag247. doi: 10.1093/ismejo/wrag247 (PMC13634200; doi:10.1093/ismejo/wrag247)
Supplement: Supplementary file 1 [file supplementary_materials_wrag247.zip › Supplementary_materials_wrag247.pdf]

Supplementary Information to:

Context-dependent siderophore exploitability shapes microbial community structure

Aileen Krüger[1,2*], Seung-Hyun Paik[3*], Michelle Bund[1*], Matthias Pesch[1*], Stephan Krueger[5], Benita Lückel[1], Athanasios Papadopoulos[6], Johanna Wiechert[1], Tanvir Hassan[4], Ulrike Weber[1,2], Romain Avellan[7], Sander Smits[6], Michael Bott[1], Ákos T. Kovács[7], Philipp Westhoff[5], Anna Matuszyńska[4], Dietrich Kohlheyer[1#], Thomas Drepper[3#], Julia Frunzke[1,2#]

[1]Institute for Bio- and Geosciences 1, IBG-1: Biotechnology, Forschungszentrum Jülich GmbH, Jülich, Germany

[2]Institute for Microbial Interactions, Faculty of Mathematics and Natural Sciences, Heinrich-Heine-University Düsseldorf, Düsseldorf, Germany

[3]Institute of Molecular Enzyme Technology, IMET, Faculty of Mathematics and Natural Sciences, Heinrich Heine University Düsseldorf, Forschungszentrum Jülich GmbH, Jülich, Germany

[4]Computational Life Science, Department of Biology, RWTH Aachen University, Aachen, Germany

[5]Plant Metabolism and Metabolomics Laboratory, Cluster of Excellence on Plant Sciences (CEPLAS), Faculty of Mathematics and Natural Sciences, Heinrich Heine University Düsseldorf, Düsseldorf, Germany

[6]Center for Structural Studies, Faculty of Mathematics and Natural Sciences, Heinrich Heine University Düsseldorf, Düsseldorf, Germany

[7]Institute of Biology, Leiden University, Leiden, Netherlands

* Contributed equally

[#]Corresponding author: j.frunzke@fz-juelich.de; t.drepper@fz-juelich.de; d.kohlheyer@fz-juelich.de

Figure S1: Full data set of plate assay on xenosiderophore utilization in *C. glutamicum.*

Figure S2: Spent media from *E. coli* is promoting growth of *C. glutamicum.*

Figure S3: Metabolomic analysis of tricarboxylic acid cycle compounds in *C. glutamicum* shows reduced citric acid production in the presence of enterobactin suggesting reduced iron limitation.

Figure S4: *In vitro* characterization of IronSenseR-mCherry.

Figure S5: *C. glutamicum* and *E. coli* duo co-cultivations demonstrate enterobactin utilization at different extents.

Figure S6: Duo co-cultivations with *P. putida* demonstrate pyoverdine as private good for *C. glutamicum* and *E. coli.*

Figure S7: *P. putida* is able to exploit enterobactin from *E. coli* WT and consequently tune down own siderophore production.

Figure S8: Growth curves and flowcytometry measurements of *E. coli*, *C. glutamicum* and *P. putida* under various iron conditions.

Figure S9: Representative single channel images of plate assays with the trio-consortium of *C. glutamicum*, *E. coli* and *P. putida*.

Figure S10: Plate assays with the trio-consortium of *C. glutamicum*, *E. coli* and *P. putida* reveals siderophore characteristics as public good and private good.

Table S1: Overview of putative siderophore-related genes in *Corynebacterium glutamicum.*

Table S2: Calculated ratio-dependent growth rates from microfluidic single-cell analysis growing in CGXII medium supplemented with 2% glucose and under iron limitation (3.6 µM FeSO4) based on cell area.

Table S3: Microbial strains used in this study.

Table S4: Plasmids used in this study.

Table S5: Primers used in this study.

Table S6: Description of all set parameters used for the modelling of microfluidic co-culture growth.

Formula S1: Derivation of conversion factor between measured cell area and volume of cells in the microfluidic system.

Method: MS-measurements of enterobactin and organic acids

Video S1: Microfluidic cultivation of *E. coli-gfp* (yellow) and *C. glutamicum-E2-crimson* (red) (Figure 3B).

Video S2: Microfluidic cultivation of *E. coli ΔentC-gfp* (yellow) and *C. glutamicum-E2-crimson* (red) (Figure 3C).

Video S3: Microfluidic cultivation of *E. coli-gfp* (yellow), *C. glutamicum-E2-crimson* (red) and *P. putida-bfp* (blue) with high abundances of *C. glutamicum* (Figure 5A).

Video S4: Microfluidic cultivation of *E. coli-gfp* (yellow), *C. glutamicum-E2-crimson* (red) and *P. putida-bfp* (blue) with high abundances of *E. coli* (Figure 5A).

Video S5: Microfluidic cultivation of *E. coli-gfp* (yellow), *C. glutamicum-E2-crimson* (red) and *P. putida-bfp* (blue) with high abundances of *P. putida* (Figure 5A).

Video S6: Microfluidic cultivation of *E. coli-gfp* (yellow), *C. glutamicum-E2-crimson* (red) and *P. putida* Δ*pvdD-bfp* (blue) with high abundances of *C. glutamicum* (Figure 5B).

Video S7: Microfluidic cultivation of *E. coli-gfp* (yellow), *C. glutamicum-E2-crimson* (red) and *P. putida* Δ*pvdD-bfp* (blue) with high abundances of *E. coli* (Figure 5B).

Video S8: Microfluidic cultivation of *E. coli-gfp* (yellow), *C. glutamicum-E2-crimson* (red) and *P. putida* Δ*pvdD-bfp* (blue) with high abundances of *P. putida* (Figure 5B).

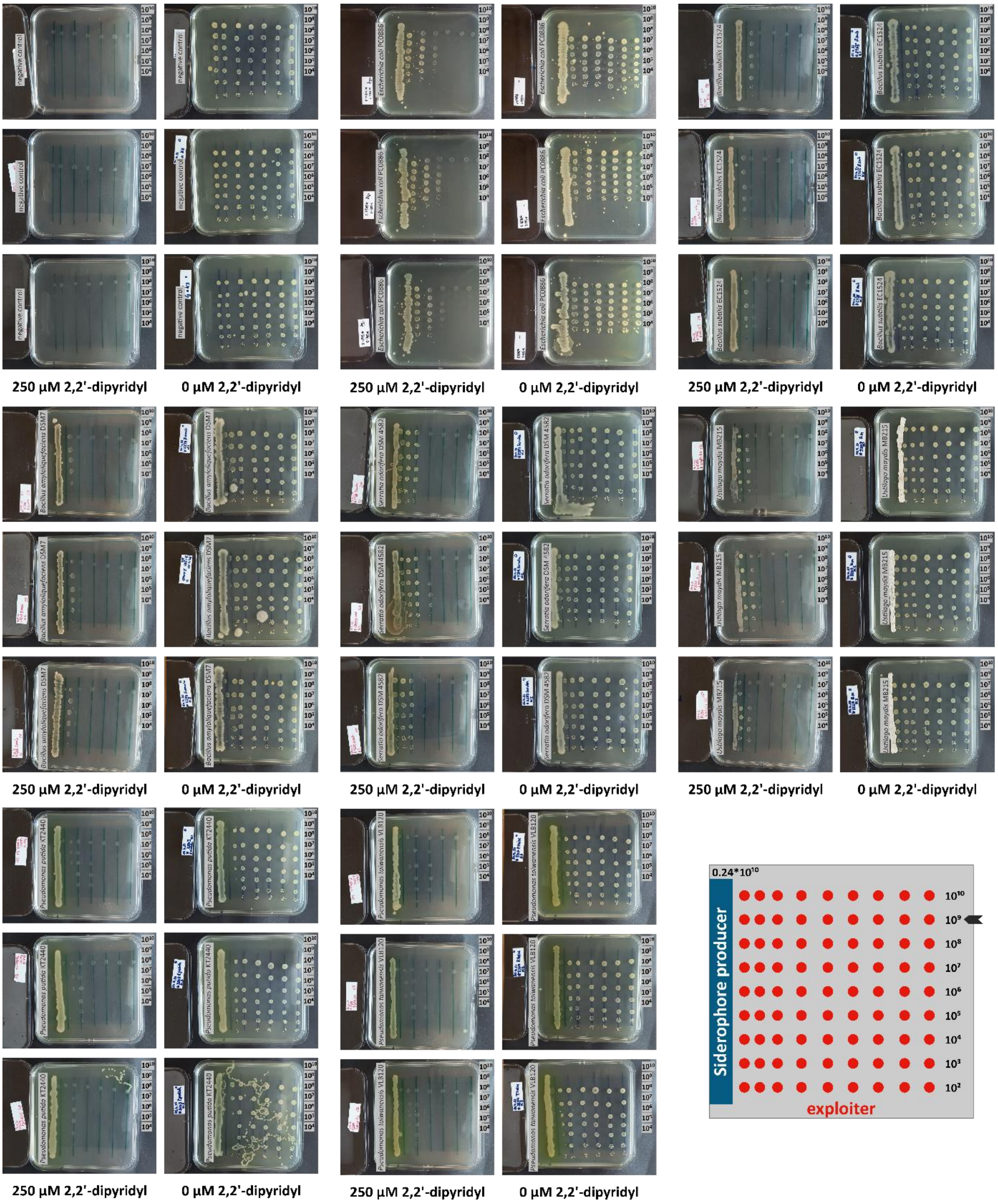

Figure S1: Full data set of plate assay on xenosiderophore utilization in *C. glutamicum*. Growth promotion of iron-starved *C. glutamicum* by seven known siderophore-producing strains was tested on double-layer LB agar containing 250 µM 2,2′-dipyridyl (DIP) as an iron chelator. Plates without chelator served as control (0 µM 2,2´-dipyridyl). *C. glutamicum* cell suspensions with different cell densities were spotted (3 µL, 10^4-10^9 cells/mL) at different distances to the pre-grown lane of siderophore producer (40 µL, $0.24*10^{10}$ cells/mL) and incubated at 30 °C for two days. The experiment was performed in independent biological duplicates or triplicates.

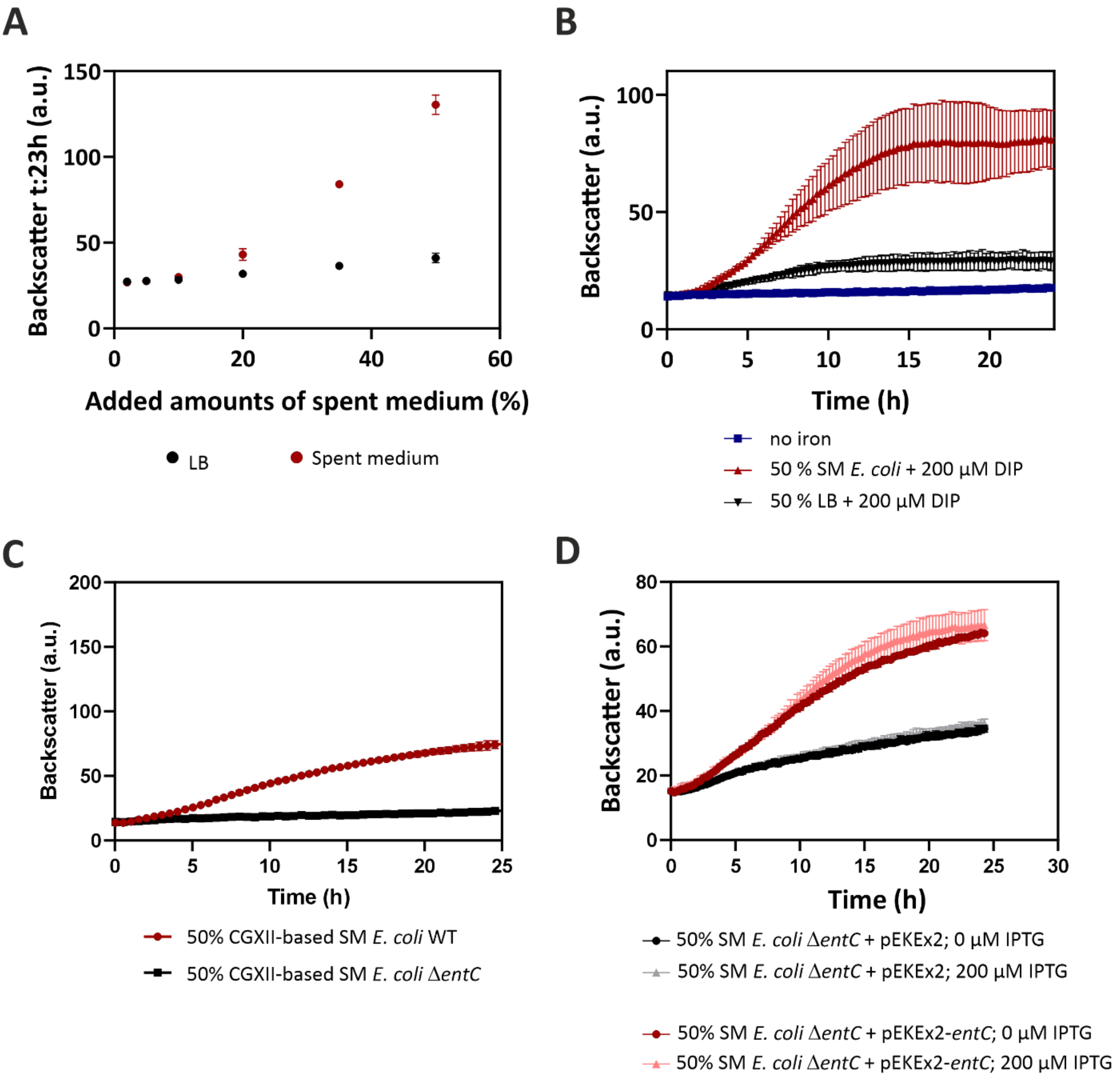

Figure S2: Spent medium from *E. coli* is promoting growth of *C. glutamicum*. Further control experiments underlining the growth-promoting effects of the spent medium (SM) from *E. coli*. **(A)** Dose-response curve at time point 23 h corresponding to growth of iron-starved *C. glutamicum* in the presence of varying amounts of LB or spent medium from *E. coli*. **(B)** Growth of *C. glutamicum* growing in the presence of 50% 2-fold CGXII supplemented with 2% glucose and without iron. Further 50% were either SM supplemented with 200 µM 2,2'-dipyridyl (DIP) (light red) or without (dark red). As control, 50% LB with 200 µM DIP (black) and also 50% CGXII without any iron (blue) were supplemented. **(C)** Comparing growth of *C. glutamicum* on SM from *E. coli* WT and the siderophore-deficient Δ*entC* variant using CGXII-based SM, instead of LB-based (Figure 2B, main text). **(D)** *E. coli* Δ*entC* strain was transformed with either empty plasmid (pEKEx2) or a plasmid harboring *entC* under the IPTG-inducible P_{tac} promoter (pEKEx2-*entC*). SM from both strains was harvested. *C. glutamicum* was cultivated in 50% 2-fold CGXII supplemented with 2% glucose and no iron, but 25 µg/mL kanamycin and either 0 or 200 µM IPTG, as well as 50% LB-SM of these respective *E. coli* strains including the *entC*-deficient (shades of black) or *entC*-overexpression (shades of red) strains. Backscatter (a.u.) values were measured in a microtiter cultivation system with *n* = 3 biological replicates.

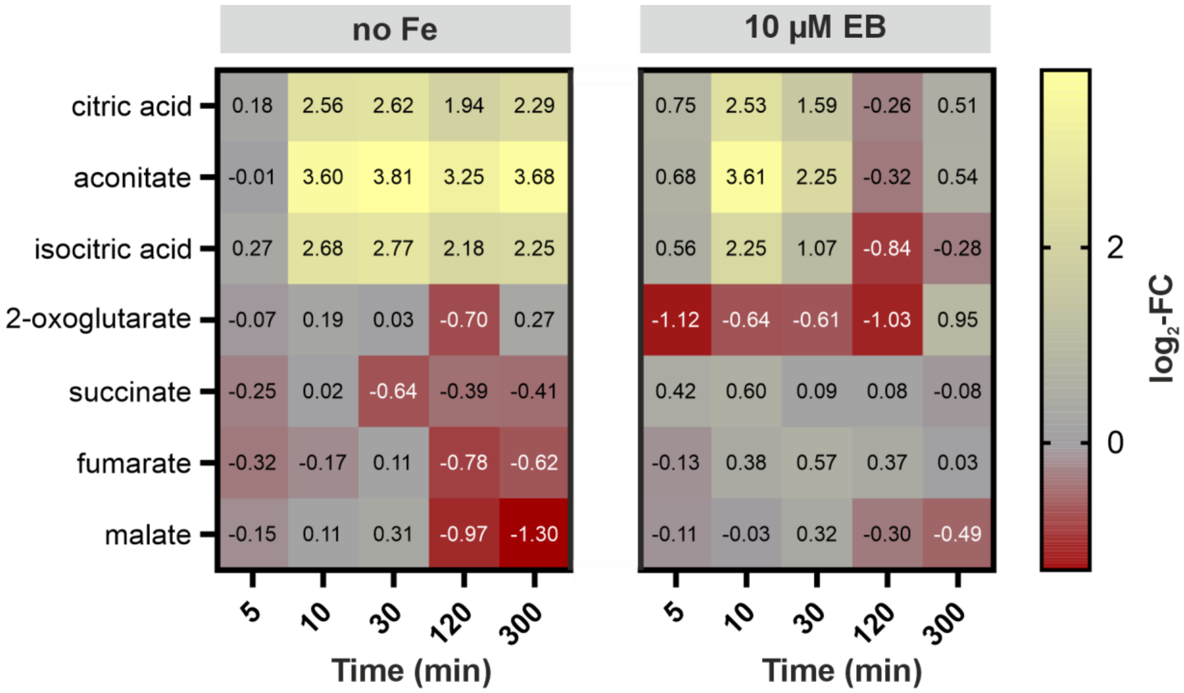

Figure S3: Metabolomic analysis of tricarboxylic acid cycle compounds in *C. glutamicum* shows reduced citric acid production in the presence of enterobactin suggesting reduced iron limitation. The heatmap represents the log_2-fold change (FC) of the normalized area (a.u.) of the MS-measurements of the no Fe or 10 µM enterobactin condition in relation to the full iron (36 µM $FeSO_4$) condition. *C. glutamicum* was cultivated in CGXII supplemented with 2% glucose and either (i) no added $FeSO_4$ but 150 µM DIP (no Fe), or (ii) 3.6 µM $FeSO_4$, 150 µM DIP and 10 µM enterobactin (10 µM EB) or (iii) 36 µM $FeSO_4$ (full iron) and no DIP (iron saturated condition). *n*= 4 biological replicates.

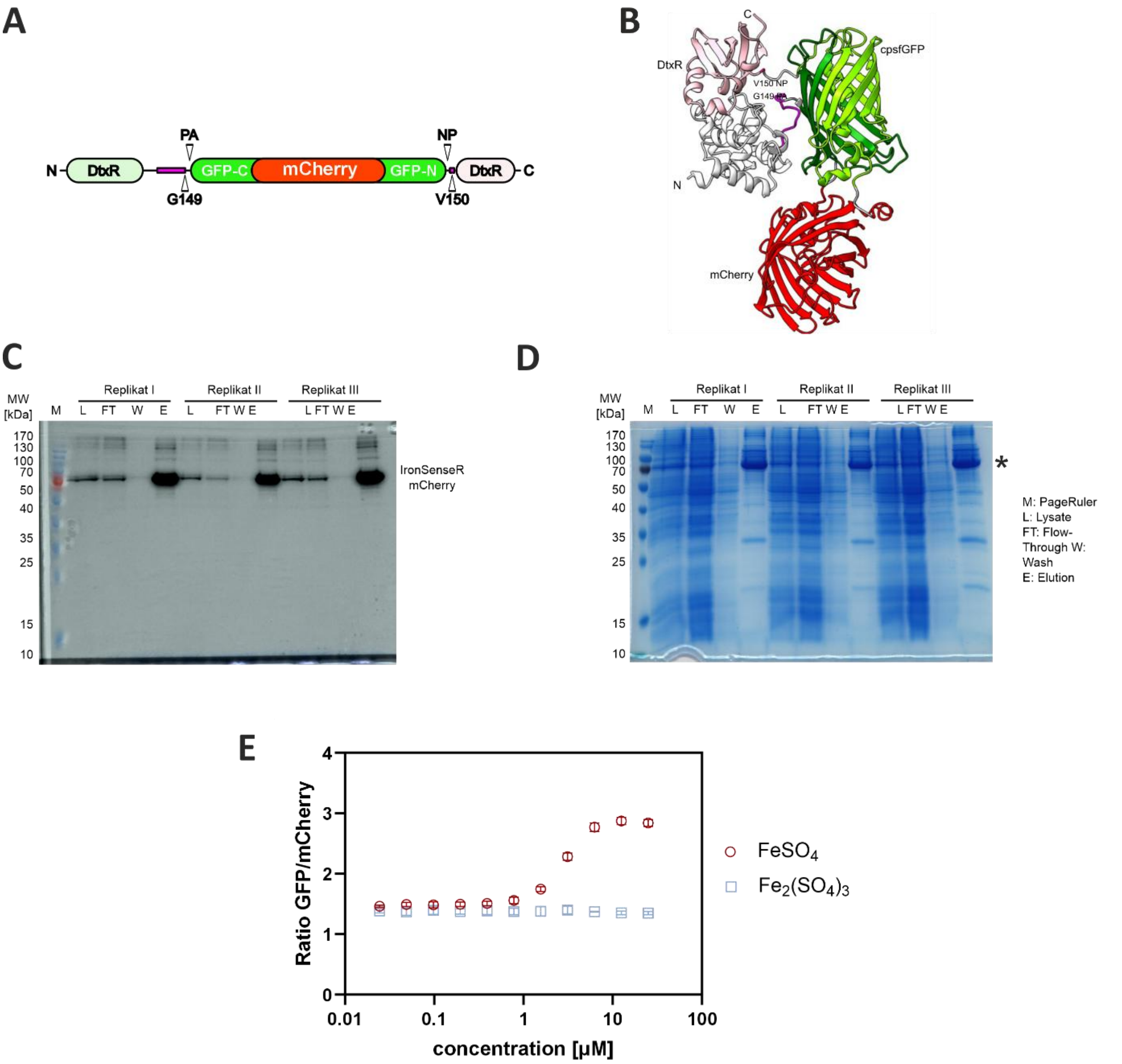

Figure S4: *In vitro* characterization of IronSenseR-mCherry. (A) Molecular architecture and **(B)** AlphaFold3 model of Matryoshka biosensor IronSenseR-mCherry for *in vivo* Fe^{2+}-detection in *C. glutamicum* and *P. putida*. According to Papadopoulos, Anlauf et al. (2026) the fluorescence intensity of the cpsfGFP domain increases with increasing Fe^{2+}-concentrations. The biosensor expression was conducted in the bacterial expression host *E. coli* and protein fractions were analyzed by SDS-PAGE after purification via affinity chromatography. **(C)** Coomassie stained images of the SDS-PAGE and **(D)** in-gel fluorescence of the SDS-PAGE of Matryoshka biosensor, IronSenseR-mCherry. The prominent band at approximately 80 kDa labeled with asterisk (*) indicates the biosensor. L: Cell lysate, FT: Flow through, W: Wash fraction, E: Elution fraction from affinity chromatography. The molecular weight is indicated in kDa. **(E)** The specificity of IronSenseR-mCherry for ferrous iron ($FeSO_4$) and ferric iron ($Fe_2(SO_4)_3$) was analyzed *in vitro*. Concentration of metal ions utilized for titration ranging from 0-25 µM. The experiments conducted in *n* = 3 biological replicates, mean values and the standard deviation (SD) is indicated. Calculated K_D-value derived from curve fit to $FeSO_4$ is 2.369 ± 0.143 µM.

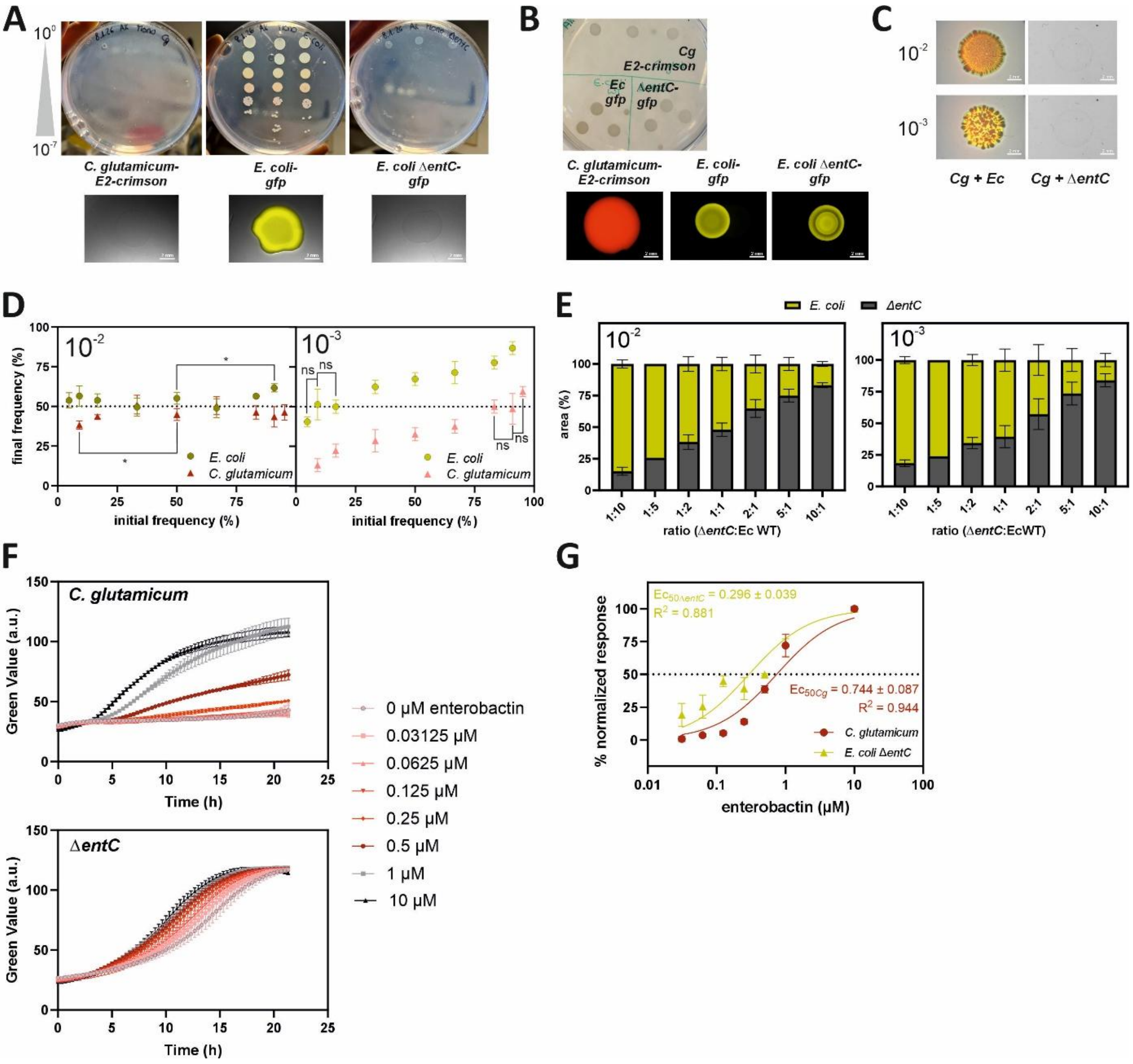

Figure S5: *C. glutamicum* and *E. coli* duo co-cultivations demonstrate enterobactin utilization at different extents. (A) Cultivation of *C. glutamicum*-E2-*crimson*, *E. coli-gfp* and *E. coli* Δ*entC-gfp* on CGXII agar plates containing 2% glucose, 3.6 µM $FeSO_4$ and 100 µM DIP in different dilutions in *n* = 3 biological replicates (top) and single drop analysis using a stereomicroscope (bottom). **(B)** Same strains in the presence of 36 µM $FeSO_4$ and no DIP, representing a sufficient iron supply. **(C)** Mixed drop (1:1) co-cultivation of *C. glutamicum* (Cg) with *E. coli* WT (Ec) or *E. coli* Δ*entC* on CGXII plates with 2% glucose, 3.6 µM $FeSO_4$ and 100 µM DIP. **(D)** Plotted final frequency vs. initial frequency for *E. coli* (yellow) and *C. glutamicum* (red) based on spot area in percentage as depicted in Figure 2I. Statistical significance was confirmed by Student's t-test ($p \leq 0.05$). **(E)** Quantified area in percentage for co-cultivation of *E. coli* WT-*gfp* (yellow) and *E. coli* Δ*entC* (grey) in varying ratios as depicted on the x-axis at two different dilutions (10^{-2} and 10^{-3}). *n* = 3 biological replicates. **(F)** Dose-response curves of *C. glutamicum* (left) and *E. coli* Δ*entC* (right) growing in CGXII supplemented with 1% glucose and 3.6 µM $FeSO_4$ as well as respective, increasing amounts of enterobactin. Growth was monitored in a shaker-incubator Growth Profiler (EnzyScreen, Netherlands). **(G)** Ec_{50} value derived from normalized growth rates (%) and a non-linear curve fit via GraphPad Prism version 10.5.0 (GraphPad Software, USA).

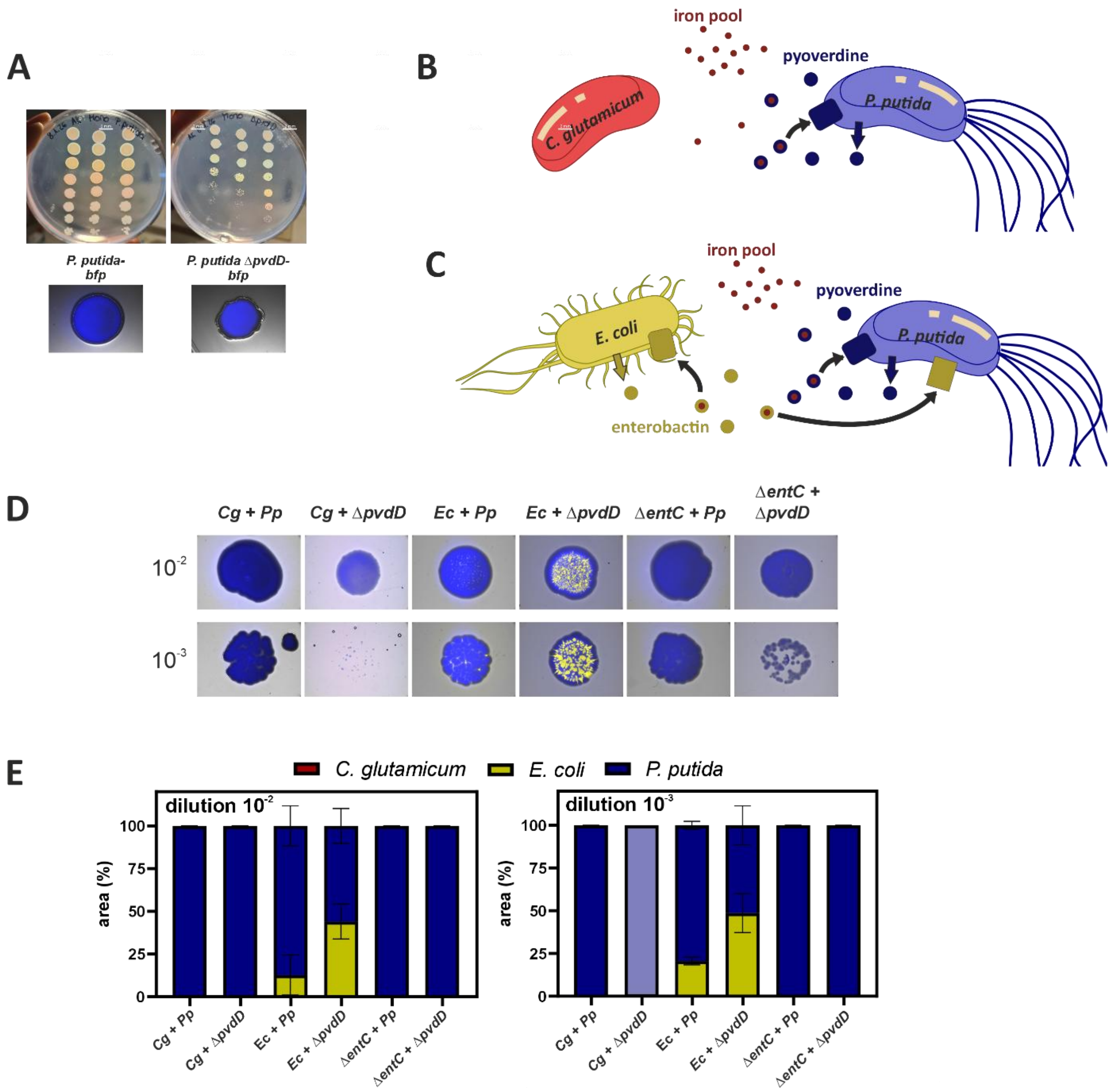

Figure S6: Duo co-cultivations with *P. putida* demonstrate pyoverdine as private good for *C. glutamicum* and *E. coli*. (A) Cultivation of *P. putida-bfp* and *P. putida ΔpvdD-bfp* on CGXII agar plates containing 2% glucose, 3.6 µM $FeSO_4$ and 100 µM DIP in increasing 10-fold dilutions in *n* = 3 biological replicates (top) and single drop analysis using a stereomicroscope (bottom). **(B)** Schematic depiction of siderophore-mediated interaction between *C. glutamicum* (non-producer) and *P. putida* (pyoverdine producer). **(C)** Schematic depiction of siderophore-mediated interaction between *E. coli* (enterobactin producer) and *P. putida* (pyoverdine producer and enterobactin exploiter, also see Figure S9). **(D)** Mixed drop (1:1) duo co-cultivation of *P. putida-bfp* (Pp) or *P. putida ΔpvdD-bfp* in all combinations with *C. glutamicum*-E2-*crimson* (Cg), *E. coli* WT-*gfp* (Ec) or *E. coli ΔentC-gfp* on CGXII plates with 2% glucose, 3.6 µM $FeSO_4$ and 100 µM DIP in two different dilutions (10^{-2} and 10^{-3}). **(E)** Quantified area in percentage for co-cultivation as shown representatively in (D). Note: Cg + *ΔpvdD* of 10^{-3} is depicted in light blue, because nearly no growth was observed (compare (D)). *n* = 3 biological replicates.

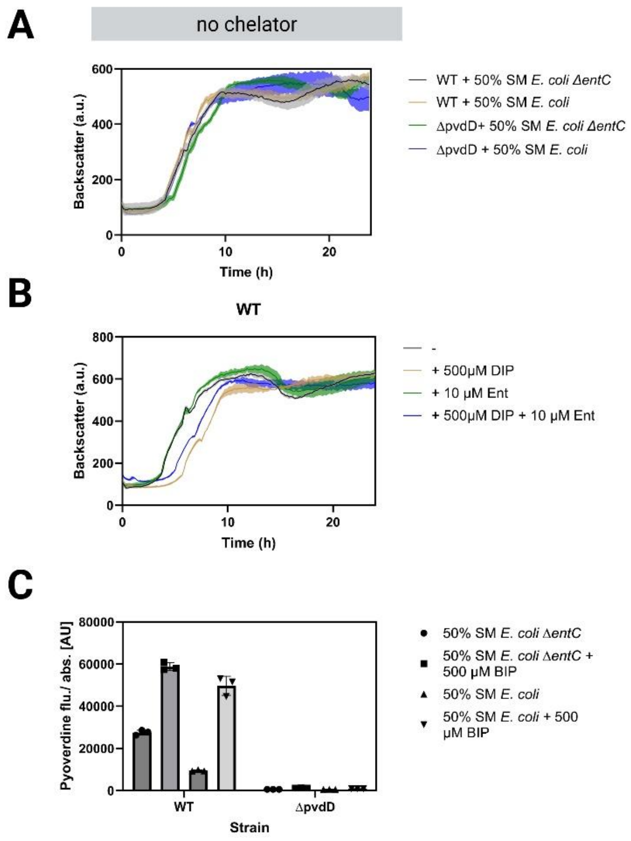

Figure S7: *P. putida* is able to exploit enterobactin from *E. coli* WT and consequently tune down own siderophore production. (A) Growth of *P. putida* WT (grey & beige) and *P. putida ΔpvdD* (green & blue) in LB with the addition of 50% *E. coli* spent medium (either from WT or Δ*entC*), with or without the addition of 500 µM DIP. **(B)** Growth of *P. putida* WT (left) and *P. putida ΔpvdD* (right) in LB with the addition of either 500 µM DIP (beige), 10 µM enterobactin (green) or both (blue). As control, LB without any additions was used (grey). **(C)** Pyoverdine fluorescence (λ_{ex} = 398 nm, λ_{em} = 455 nm) of cultures after cultivation with *E. coli* spent media.

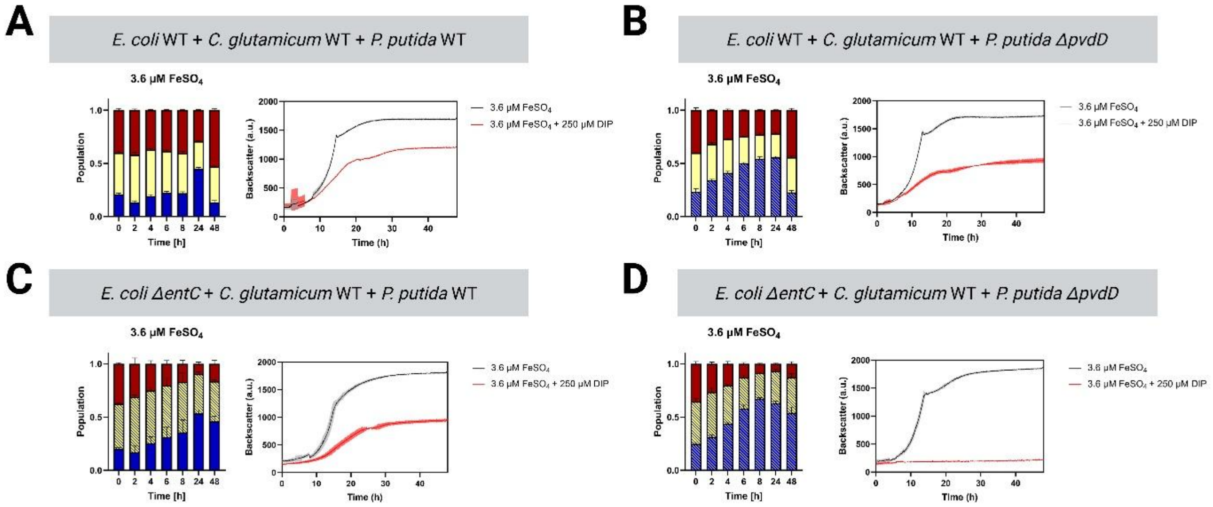

Figure S8: Growth curves and flow cytometry measurements of *E. coli*, *C. glutamicum* and *P. putida* under various iron conditions. (A-D) Bar charts showing populations of trio cultures grown in CGXII (2% glucose + 3.6 µM $FeSO_4$) measured via flow cytometry on the left and growth curves of the same cultures as well as the + DIP control on the right. Red bars represent *C. glutamicum*, yellow bars represent *E. coli*, striped yellow bars represent *E. coli ΔentC*, blue bars represent *P. putida*, and striped blue bars represent *P. putida ΔpvdD*.

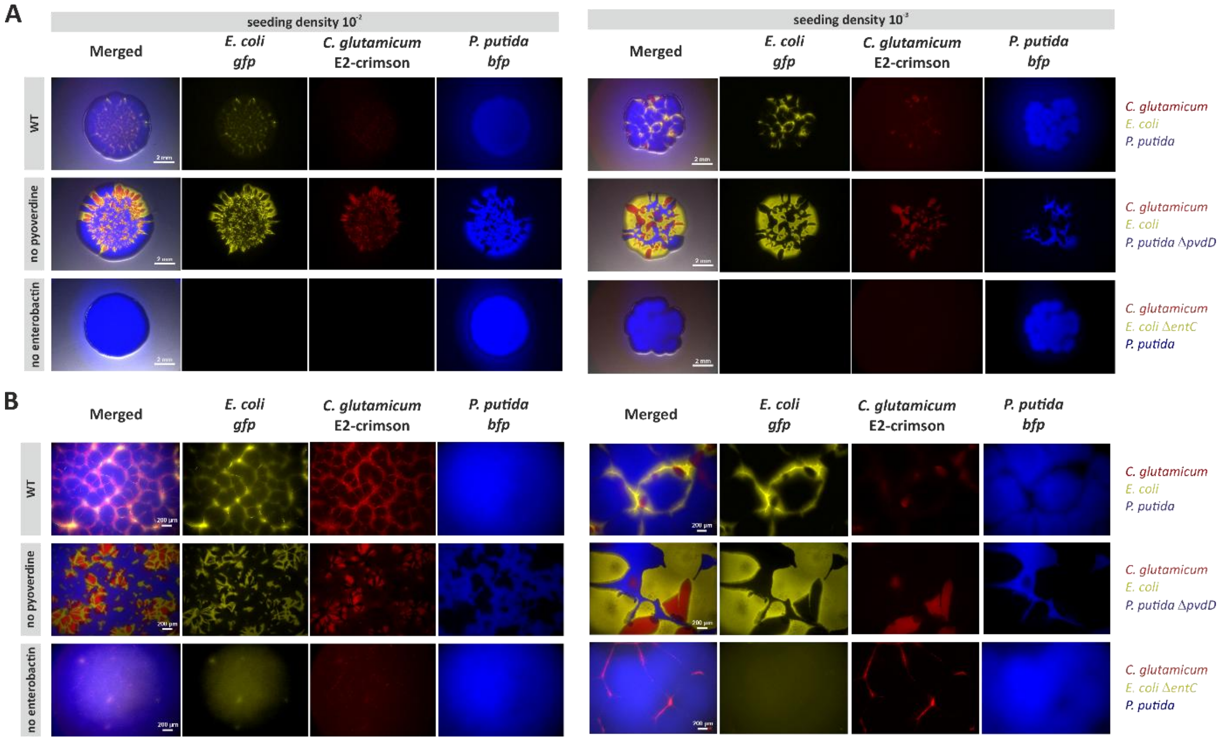

Figure S9: Representative single channel images of plate assays with the trio-consortium of *C. glutamicum*, *E. coli* and *P. putida*. Single channel images for the representative stereomicroscopy pictures of mixed drop assay on iron-restricted plates as shown in Figure 4G with *C. glutamicum-E2-crimson* (red), *E. coli-gfp* (yellow) and *P. putida-bfp* (blue), as well as siderophore-deficient mutants, as indicated in two different seeding densities in a 1:1:1 ratio and **(A)** 16x vs. **(B)** 80x zoom.

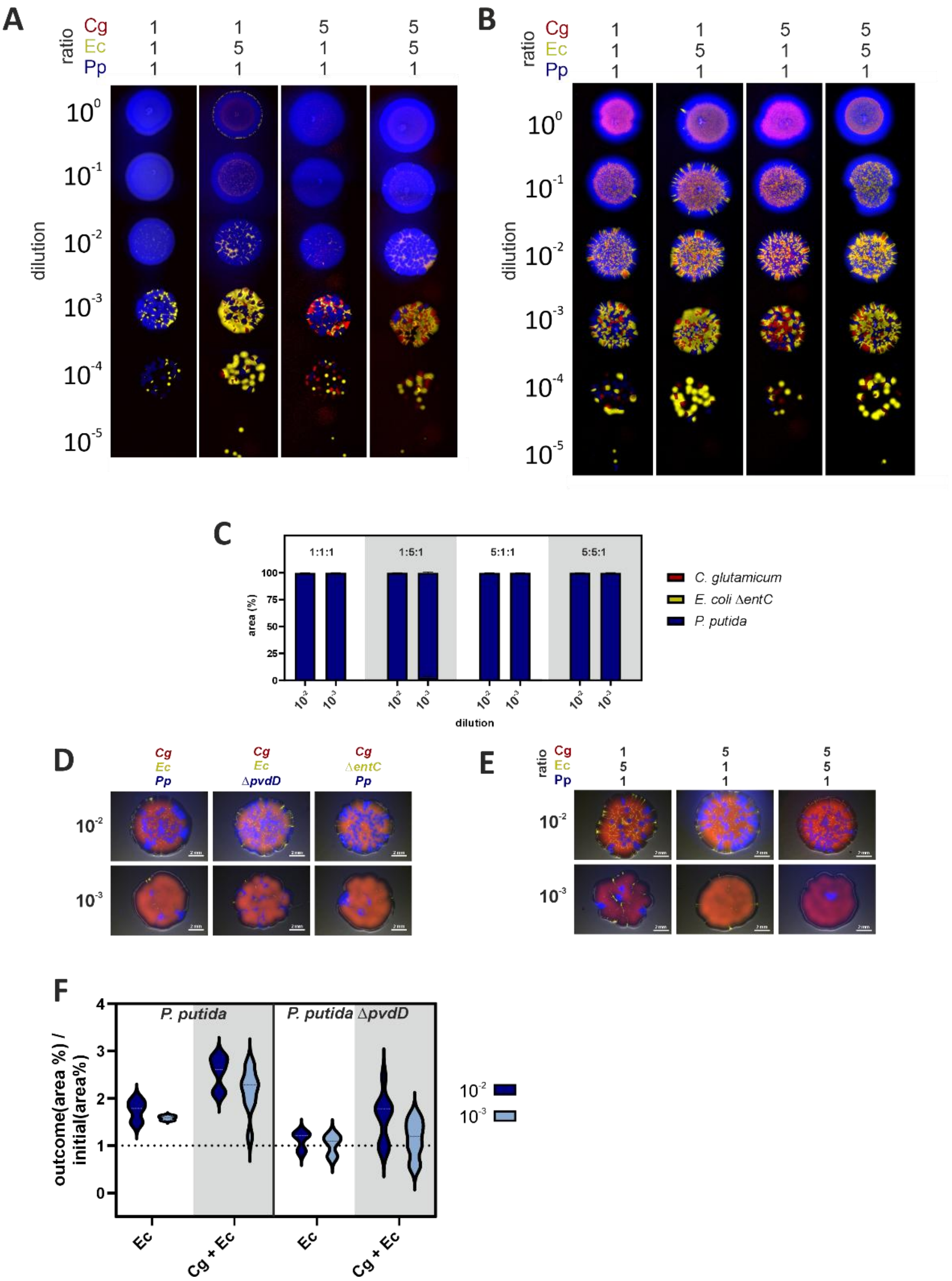

Figure S10: Plate assays with the trio-consortium of *C. glutamicum*, *E. coli* and *P. putida* reveals siderophore characteristics as public good and private good. (A) Mixed drop trio co-cultivation of *P. putida-bfp* (Pp) or **(B)** *P. putida* Δ*pvdD-bfp* with *C. glutamicum*-E2-*crimson* (Cg) and *E. coli* WT-*gfp* (Ec), in different ratios as depicted on top, on CGXII plates with 2% glucose, 3.6 µM $FeSO_4$ and 100 µM DIP in different dilutions (10^0 to 10^{-5}). **(C)** Quantified area in percentage for no-enterobactin trio co-cultivation of *C. glutamicum*, *E. coli* Δ*entC-gfp* and *P. putida* at varying ratios in two different dilutions (10^{-2} and 10^{-3}) on iron-limited CGXII agar plates. *n* = 3 biological replicates. **(D)** Trio-cultivations of Cg-Ec-Pp and the siderophore-deficient variants on CGXII agar plates with 2% glucose, 36 µM $FeSO_4$ and PCA as siderophore-independent iron excess control. **(E)** WT variants in different ratios at iron excess control. One representative of *n* = 3 biological replicates. **(F)** *P. putida* WT and Δ*pvdD* outcome analysis with a ratio of *P. putida* outcome over initial area in percentage for two seeding densities (10^{-2} and 10^{-3}) in duo and trio co-cultivations.

Table S1: Overview of putative siderophore-related genes in *Corynebacterium glutamicum*.

Locus	Start	Stop	Annotation	up-/down-regulated in response to iron limitation*
NCgl0036	37245	38201	putative iron-siderophore ABC transporter, permease protein	down
NCgl0037	38202	38981	putative iron-siderophore ABC transporter, ATP-binding protein	
NCgl0038	38978	39802	putative iron-chelator utilization protein	
NCgl0329	352693	353640	putative ironIII dicitrate ABC transporter, secreted siderophore-binding lipoprotein	up
NCgl0411	448127	449179	putative spermidine/putrescine/ironIII ABC transporter, ATPase subunit	down
NCgl0412	449180	450826	putative spermidine/putrescine/ironIII ABC transporter, permease subunit	
NCgl0413	450834	451913	putative spermidine/putrescine/ironIII ABC transporter, substrate-binding lipoprotein	
NCgl0482	528776	529570	putative siderophore ABC transporter, ATP-binding protein	up
NCgl0483	529589	530755	putative siderophore ABC transporter, permease protein	
NCgl0484	530745	531791	putative siderophore ABC transporter, permease protein	
NCgl0618	659543	660541	putative iron-siderophore ABC transporter, substrate-binding lipoprotein	up
NCgl0635	680128	681027	putative cytoplasmic siderophore-interacting protein	up
NCgl0636	681037	681846	putative iron-siderophore ABC transporter, ATP-binding protein	
NCgl0637	681843	682871	putative iron-siderophore ABC transporter, permease subunit	
NCgl0638	682868	683863	putative iron-siderophore ABC transporter, permease subunit	
NCgl0639	683873	684925	putative iron-siderophore ABC transporter, secreted siderophore-binding lipoprotein	
NCgl0644	688913	689890	putative iron-siderophore ABC transporter, substrate-binding lipoprotein	n.d.
NCgl0645	689914	690696	putative iron-siderophore ABC transporter, ATPase subunit	
NCgl0646	690703	691722	putative iron-siderophore ABC transporter, permease subunit	
NCgl0773	848496	849323	putative cytoplasmic siderophore-interacting protein	up
NCgl0774	849323	850243	putative secreted siderophore-binding lipoprotein	

NCgl0776	851351	852367	putative iron-siderophore ABC transporter, substrate-binding lipoprotein	up
NCgl0777	52618	853619	putative iron-siderophore ABC transporter, permease subunit	up
NCgl0778	853612	854727	putative iron-siderophore ABC transporter, permease subunit	up
NCgl0779	854724	855479	putative iron-siderophore ABC transporter, ATPase subunit	up
NCgl1200	1313270	1314121	putative cytoplasmic siderophore-interacting protein	up
NCgl1209	1322393	1323409	putative secreted siderophore-binding lipoprotein	up
NCgl1395	1529486	1530223	putative cytoplasmic siderophore-interacting protein	n.d.
NCgl1564	1721780	1722856	putative iron-siderophore ABC transporter, substrate-binding lipoprotein	n.d.
NCgl1565	1722870	1723829	putative iron-siderophore ABC transporter, ATPase subunit	n.d.
NCgl1566	1723826	1724581	putative iron-siderophore ABC transporter, permease subunit	n.d.
NCgl1905	2089215	2089868	phosphopantetheinyl transferase, iron-chelating complex subunit	n.d.
NCgl1959	2148225	2149169	putative ironIII dicitrate ABC transporter, substrate-binding lipoprotein	up
NCgl2031	2229096	2229896	putative ironIII dicitrate ABC transporter, ATPase subunit	n.d.
NCgl2032	2229897	2230937	putative ironIII dicitrate ABC transporter, permease subunit	n.d.
NCgl2033	2230944	2231933	putative ironIII dicitrate ABC transporter, substrate-binding lipoprotein	n.d.
NCgl2970	3284397	3285425	Putative ironIII dicitrate ABC transporter, substrate-binding lipoprotein	up

*(Küberl, Mengus-Kaya et al. 2020), comparison of low (1 µM) and high (36 µM) $FeSO_4$ levels, genes with a ≥ 2-fold altered mRNA ratio and a ρ value of ≤ 0.05.

Table S2: Calculated ratio-dependent growth rates from microfluidic single-cell analysis growing in CGXII medium supplemented with 1% glucose and under iron limitation (3.6 µM $FeSO_4$) based on cell area.

Culture type	Organism	Partner 1	Partner 2	µ ± SD (h^{-1})	*N* (chambers)
Mono	*C. glutamicum* WT	–	–	0.01 ± 0.01	26
Mono	*E. coli* WT	–	–	0.26 ± 0.03	45
Mono	*E. coli* Δ*entC*	–	–	0.21 ± 0.02	24
Mono	*P. putida* WT	–	–	0.35 ± 0.02	12
Mono	*P. putida* Δ*pvdD*	–	–	0.27 ± 0.01	10
Co	*C. glutamicum* WT	*E. coli* WT	–	0.29 ± 0.03	24
Co	*E. coli* WT	*C. glutamicum* WT	–	0.23 ± 0.03	24
Co	*C. glutamicum* WT	*E. coli* Δ*entC*	–	0.03 ± 0.01	30
Co	*E. coli* Δ*entC*	*C. glutamicum* WT	–	0.17 ± 0.02	30
Co	*E. coli* WT	*P. putida* WT	-	0.14 ± 0.08	34
Co	*P. putida* WT	*E. coli* WT	-	0.32 ± 0.03	34
Co	*E. coli* WT	*P. putida* Δ*pvdD*	-	0.20 ± 0.08	32
Co	*P. putida* Δ*pvdD*	*E. coli* WT	-	0.18 ± 0.10	32
Co	*E. coli* Δ*entC*	*P. putida* WT	-	0.00 ± 0.13	22
Co	*P. putida* WT	*E. coli* Δ*entC*	-	0.33 ± 0.03	22
Co	*E. coli* Δ*entC*	*P. putida* Δ*pvdD*	-	0.05 ± 0.05	42
Co	*P. putida* Δ*pvdD*	*E. coli* Δ*entC*	-	0.14 ± 0.07	42
Tri	*C. glutamicum* WT	*E. coli* WT	*P. putida* WT	0.14 ± 0.08	36
Tri	*E. coli* WT	*C. glutamicum* WT	*P. putida* WT	0.20 ± 0.10	36
Tri	*P. putida* WT	*E. coli* WT	*C. glutamicum* WT	0.26 ± 0.12	36
Tri	*C. glutamicum* WT	*E. coli* WT	*P. putida* Δ*pvdD*	0.16 ± 0.09	26
Tri	*E. coli* WT	*C. glutamicum* WT	*P. putida* Δ*pvdD*	0.25 ± 0.02	26
Tri	*P. putida* Δ*pvdD*	*E. coli* WT	*C. glutamicum* WT	0.22 ± 0.07	26

Table S3: Microbial strains used in this study.

Strain	Relevant characteristics	Reference	Purpose
Corynebacterium glutamicum			
ATCC 13032	Biotin-auxotrophic wild type	DSMZ culture collection: DSM20300 (Kinoshita, Udaka et al. 2004)	*C. glutamicum* WT, siderophore exploiter
WT::cg1121/22_P_{tet} *crimson*	ATCC 13032 derivative with an inserted *e2-crimson* gene under control of the constitutive P_{tet} promoter (-*tetR*, constitutive), integrated in the cg1121/22 intergenic region	This study	*C. glutamicum* WT, E2-crimson-labeled
Escherichia coli			
DH5α	F⁻⁻ *supE44* Δ*lacU169* (ϕ80*lacZ*DM15) *hsdR17 recA1 endA1 gyrA96 thi-1 relA1*	Invitrogen	Cloning procedures
E. coli Pir2	F - Δlac169 rpoS (Am) robA1 creC510 hsdR514 endA reacA1 uidA (ΔMlui)::pir	Life Technologies	Cloning of plasmids with R6K ori
E. coli HB101	F – mcrB mrr hsdS20(rB– mB–) recA13 leuB6 ara-14 proA2 lacY1 galK2 xyl-5 mtl-1 rpsL20(SmR) gln V44λ–	Boyer and Roulland-Dussoix (1969)	Conjugation
PC 0886	$lambda^-$ F^- sup^-	DSMZ culture collection: DSM 13127	*E. coli* WT, siderophore producer
PC 0886 Δ*entC*	PC 0886 derivative with in-frame deletion of the *entC* gene $lambda^-$ F^- sup^- Δ*entC*	This study	Non-siderophore producer
PC 0886 *nupG*::*neoR*_ oopT_P_{J23150} - *gfpmut1*	PC 0886 derivative with an inserted *neoR* and *gfpmut1* gene under control of P_{J23150}, separated by oopT and integrated behind the *nupG* locus	This study	*E. coli* WT, siderophore producer, Gfpmut1-labeled
PC 0886 Δ*entC* *nupG*::*neoR*_ oopT_P_{J23150} - *gfpmut1*	PC 0886 Δ*entC* derivative with an inserted *neoR* and *gfpmut1* gene under control of P_{J23150}, separated by oopT and integrated behind the *nupG* locus	This study	*E. coli* Δ*entC*, non-siderophore producer, Gfpmut1-labeled
Pseudomonas putida			
KT2440	Wild-type *P. putida*, Pyoverdine producer	ATCC culture collection: ATCC 47054 (Nelson, Weinel et al. 2002)	Siderophore producer

Δ*pvdD*	*P. putida* KT2440 with in-frame deletion of *pvdD* gene	Paik et al. unpublished	Non-siderophore producer
KT2440-bfp	*P. putida KT2440* with genomic attTn7 integration of: P_{em7} *mTagBFP, GmR*	This study	*P. puitda* WT, siderophore producer, mTagBFP-labeled
Δ*pvdD-bfp*	*P. putida* KT2440 Δ*pvdD* with genomic attTn7 integration of: P_{em7} *mTagBFP, GmR*	This study	*P. puitda* KT2440 Δ*pvdD* siderophore producer, mTagBFP-labeled
Δ*pvdD*-IronSenseeR-mCherry	*P. putida* KT2440 Δ*pvdD* with genomic attTn7 integration of: *IronSensR-mCherry, GmR*	This study	*P. puitda* KT2440 Δ*pvdD* siderophore non-producer, with IPTG inducible IronSenseR-mCherry expression
**Other microorganisms**			
Serratia odorifera ATCC 33077	Wild-type *S. odorifera*, catechol-type siderophore producer	DSMZ culture collection: DSM 4582	Siderophore producer
Bacillus subtilis EC1524	Wild-type *B. subtilis*, catechol-type siderophore producer	John Innes Centre, Norwich, Great Britain	Siderophore producer
Bacillus amyloliquefaciens ATCC 23350	Wild-type *B. amyloliquefaciens*, catechol-type siderophore producer	DSMZ culture collection: DSM7	Siderophore producer
Pseudomonas taiwanensis VLB120	Wild-type *P. taiwanensis*, mixes-type siderophore producer; styrene prototroph	(Panke, Witholt et al. 1998)	Siderophore producer
Ustilago maydis MB215	Wild-type *U. maydis*, hydroxamate-type siderophore producer	DSMZ culture collection: DSM17144	Siderophore producer

Table S4: Plasmids used in this study.

Plasmid	Relevant characteristics	Reference
pJC1	*Kan*^{R}, *Amp*^{R}; *oriV*$_{C.g.}$, *oriV*$_{E.c.}$<br>*C. glutamicum*/*E. coli* shuttle vector	(Cremer, Eggeling et al. 1990)
pJC1-*venus*-term	*Kan*^{R}, *oriV*$_{C.g.}$, *oriV*$_{E.c.}$<br>pJC1 derivative carrying the *venus* coding sequence, followed by a *Bacillus subtilis* terminator sequence	(Baumgart, Luder et al. 2013)
pJC1-Δ*entC*_disruption-cassette	*Kan*^{R}, *oriV*$_{C.g.}$, *oriV*$_{E.c.}$<br>pJC1 derivative carrying the disruption cassette for in-frame deletion of *entC*, consisting of approximately 200 nt homologous up- and downstream regions flanking the *neoR* gene	This work
pKD13	*Amp*^{R}, *Kan*^{R}, *oriR*$_{6K\gamma}$, *tL3LAM*, *rgnB*, *neoR*<br>Template plasmid for the amplification of *neoR* gene flanked by the frt sites (disruption cassette)	(Datsenko and Wanner 2000)
pKD46	*Amp*^{R}, *oriR*$_{101}$, *repA101ts*, *araC*, *bet*, *gam*, *exo*<br>Temperature sensitive plasmid for L-arabinoase-inducible expression of the lambda red recombinase system	(Datsenko and Wanner 2000)
pCP20	*Amp*^{R}, Cat^R, *ts-rep*, *cl857*, *flp*<br>Temperature sensitive plasmid for constitutive expression of FLP recombinase	(Cherepanov and Wackernagel 1995)
pJC1-*nupG* LF-BcuI-*nupG* RF_1S	*Kan*^{R}, *oriV*$_{C.g.}$, *oriV*$_{E.c.}$<br>pJC1 derivative carrying the left and right homologous regions (200-230 nt) of the integration site downstream of *nupG*, separated by a BcuI site.<br>Helper plasmid for reporter integration in *E. coli* no. 1.	This work
pJC1-*nupG* LF-*neoR*-oopT-BcuI-*nupG* RF_2S	*Kan*^{R}, *oriV*$_{C.g.}$, *oriV*$_{E.c.}$<br>pJC1-*nupG* LF-BcuI-*nupG* RF_1S derivative with an inserted *neoR*-oopT and a restored BcuI site.<br>Helper plasmid for reporter integration in *E. coli*	This work
pJC1-nupG LF-*neoR*-oopT-P$_{J23150}$-*gfpmut1*-*nupG* RF_3S	*Kan*^{R}, *oriV*$_{C.g.}$, *oriV*$_{E.c.}$<br>pJC1 derivative carrying the integration cassette for insertion of the *neoR* and P$_{J23150}$-*gfpmut1* sequence, separated by the oopT terminator.<br>P$_{J23150}$-*gfpmut1*-specific plasmid for integration in *E. coli*.	This work
pK18-*mobsacB*-cg1121/22-Lrp-sensor	*Kan*^{R}, *oriV*$_{E.c.}$, *sacB*, *lacZα*<br>Plasmid for genomic integration of the Lrp-sensor in the intergenic region of cg1121-cg1122 in *C. glutamicum*.	(Mustafi, Grünberger et al. 2014)
pK19-*mobsacB*	*Kan*^{R}, *oriV*$_{E.c.}$, *sacB*, *lacZα*<br>Plasmid for allelic exchange in *C. glutamicum*	(Schäfer, Tauch et al. 1994)
pSM24	*kan*^{R}, pCLTON1^{TS} (RepA^{P47S}) (#d) derivative containing the *dcas9* gene	Gift from S. Matamouros
pK19-*mobsacB*-cg1121/22_P$_{tet}$-*e2-crimson*	*Kan*^{R}, *oriV*$_{E.c.}$, *sacB*, *lacZα*<br>pK19-*mobsacB* derivative for inserting the e2-crimson gene under control of the constitutive P$_{tet}$ promoter (-*tetR*, constitutive) in the cg1121/22 intergenic region	This work
pPREx2-MDtxR-GA-G149	Kan^r, pPREx2 derivative carrying the matryoshka cassette, encoding for the cpsfGFP and nested LSSmApple inserted into the respective insertion sites of DtxR (I138-V150) under the control of P$_{tac}$ promoter	(Papadopoulos, Anlauf et al. 2026)
pK19*mobsacB*-*strep-mcherry-dps*	Kan^r, pK19*mobsacB* derivative derivative encoding for the fusion protein mCherry-Dps	Sundermeyer, unpublished
pET24b-NHis	Kan^r,	Hentschel, unpublished
pPREx2-10xHis-TEV-MDtxR-GC-G149	Kan^r, pPREx2 derivative carrying the matryoshka cassette, encoding for the cpsfGFP and nested mCherry inserted into the	This work

	respective insertion sites of DtxR (I138-V150) with an N-terminal 10xHis tag and TEV cleavage site under the control of P_{tac} promoter	
pRK2013	*Kan*^{r}, *oriV*(RK2/ColE1) -mob+ tra+	Figurski and Helinski (1979)
pTNS1	*Amp*^{R}, *oriV*(R6K), *TnSABC+D* operon	Choi, Gaynor et al. (2005)
mTn7_mCherry	*Kan*^{r}, Gm^r, *oriV* (R6K), pBG-derived, promoter Pem7, *mCherry*	Paik et al. unpublsihed
mTn7_mTagBFP	*Kan*^{r}, Gm^r, *oriV* (R6K), pBG-derived, promoter Pem7, *mTagBFP*	This study
mtn7_Ptac_IronSensR	*Kan*^{r}, Gm^r, *oriV* (R6K)*, Ptac, lacI, IronSensR*	Papadopoulos, Anlauf et al. (2026)
mtn7_Ptac_IronSensR-mCherry	*Kan*^{r}, Gm^r, *oriV* (R6K), *Ptac, lacI, IronSensR-mCherry*	This study

Table S5: Primers used in this study.

#	Sequence (5´-3´)	Purpose
**Primers for plasmid construction**		
1145	AGCGACGCCGCAGGGGGATCCGGCGCAGGACATCACATTGCG	Construction of pJC1-Δ*entC*_disruption-cassette
1146	CGAAGCAGCTCCAGCCTACAATCATCCTCCACAAAATGATAAAGGC	
1147	TGTAGGCTGGAGCTGCTTCG	Construction of pJC1-Δ*entC*_disruption-cassette, sequencing of the genomic *entC* deletion
1148	CGGGGATCCGTCGACCTG	Construction of pJC1-Δ*entC*_disruption-cassette, sequencing of final plasmid
1149	CAGGTCGACGGATCCCCGCATGTTGAACGTTTTTGGATTGCATTAA	Construction of pJC1-Δ*entC*_disruption-cassette
1150	AAAACGACGGCCAGTACTAGCTACACGCGAGGTTATCCGC	
1112	AGCGACGCCGCAGGGGGATCCGATGTTCCTGATGATGACTAACGG	Construction of pJC1-*nupG* LF-BcuI-*nupG* RF_1S
1114	CGCAAAGAAAAACGGGTCGCC	
1115	AAAACGACGGCCAGTACTAGGGCGTGCTTTGCGTGGTACC	
1604	GGCGACCCGTTTTTCTTTGCGACTAGTTAATTAGTGGCTAACCGTCTG TGTG	
1633	GTGCACAAGGGATGTTCCTGATG	
1634	GTTGGTGCGGATTCGTGATGC	
1605	GACGGTTAGCCACTAATTAACTAGGTGTAGGCTGGAGCTGCTTC	Construction of pJC1-*nupG* LF-*neoR*-oopT-BcuI-*nupG* RF_2S
1615	GACCCGTTTTTCTTTGCGACTAGACTAGTAAAAAACGCCCGGCGGCA ACCGAGCGTTCTGAATTACTTAGTCTAGCTTACCTTAGTTCCTATTCC GGATCCGTCGACCTGCAGTT	
1629	CCCGTTTTTCTTTGCGACTAGACTAGCTATTTTTTGTATGGTTCATCCA TGCCATG	Construction of pJC1-nupG LF-*neoR*-oopT-P_{J23150}-*gfpmut1*-*nupG* RF_3S
1630	CGGTTGCCGCCGGGCGTTTTTTACTAGTTTACGGCTAGCTCAGTCCTA GGTATTATACTAGTTGAACTTTAAGAAGGAGATATCATATGGGTACC CTGCAGATGAGTAAAG	
1592	CAAACTGTCATTGCCATACGCGAATTCGTTGAACTAATGGGTGCTTTA GTTGAAG	Construction of pK19-*mobsacB*-cg1121/22_P_{tet}-*e2-crimson*
1593	TGATATCTCCTTCTTAAAGTTCATGCAGGTGTATCAACAAGCTGGG	
259	TGAACTTTAAGAAGGAGATATCATATGGATAGCACTGAGAACGTCAT C	
1595	CTATTTAAGAAGTTTAAATTGTGTCCATGAGTTCGCTCGACTACTGGA ACAGGTGGTGG	
264	cacaagcttaattaaGgaattcgagctcgg	Constuction of mTn7_mTagBFP
265	aatcagctcgctcattagaaaacctccttagcatg	
266	taaggaggttttctaatgagcgagctgattaagg	
267	ccgagctcgaattcCttaattaagcttgtgcccc	
292	CTGTACGGCGGCACCatggtgagcaagggc	Construction of mtn7_Ptac_IronSensR-mCherry
293	GCTGGCGCTGCCGCCcttgtacagctcgtccat	
294	gacgagctgtacaagGGCGGCAGCGCCAGC	
295	gcccttgctcaccatGGTGCCGCCGTACAG	
**Construction of pPREx2-MDtxR-GC-G149 (IronSenseR-II)**		
Matr-S208 fw	CAAGTTGGACATCACCTCCCACAACGAGGACTAC	Construction of IronSenseR-mCherry
pPREx2 rv	ATGTATATCTCCTTCTGCAGGC	
10xHisTEV fw	ATGCCTGCAGAAGGAGATATACATATGAGCAGCCATCATCATC	
TEV rv	CGGTGGTATCGACCAGATCCTTCATGCTAGCGCCCTGAAAATAC	
Matr-adj fw	ATGAAGGATCTGGTCGATAC	

Matr rv	ATCCTCCTCGCCCTTGCTCACGGTGCCGCCATACAGTTC	
mCherry fw	ACTGTATGGCGGCACCGTGAGCAAGGGCGAGGAG	
mCherry-short rv	AGTCCTCGTTGTGGGAGGTGATGTCCAACTTGATG	
**Confirmation of integration by colony-PCR**		
Tn7R	CACAGCATAACTGGACTGATTTC	Choi and Schweizer (2006)
PglmS-down	GCACATCGGCGACGTGCTCTC	
Seq1-MDtxR-GC-G149 fw	GCCGACATCATAACGGTTCTGG	Sequencing primers for IronSenseR-mCherry
Seq2-MDtxR-GC-G149 rv	CTTGTACAGCTCGTCCATGCCGC	
Seq3-MDtxR-GC-G149 rv	AGACCGCTTCTGCGTTCTG	
**Universal primers for plasmid sequencing**		
R12	CAGGGACAAGCCACCCGCACA	Universal sequencing primers of pJC1-based plasmids
R13	GGAAGCTAGAGTAAGTAGTTCGC	
920	CATCGGCTCGTATAATGTGTGG	Universal sequencing primers of pEKEx2-based plasmids
891	GAAAATCTTCTCTCATCCGCC	
M19	CGCCAGGGTTTTCCCAGTCAC	Universal sequencing primers of pK19-*mobsacB*-based plasmids
M20	AGCGGATAACAATTTCACACAGGA	
**Primers for sequencing of genomic insertions/deletions**		
89	CGCGCGATAAATGTGCGTTTGAG	Sequencing of the genomic insertions at integration locus cg1121/22
90	CAAGGCCTAGTGTTCACTCAGG	
921	GTACCAGCGCGGTTTCACCAG	Sequencing of the genomic *entC* deletion
922	CGACATGCTGCGAGTGACAGC	
633	CAGGTCGACTCTAGAGGATCCAGAAGTTCGCTCAGCACCTTC	Sequencing of the P_{J23150}-*gfpmut1* insertion downstream of *nupG*
1148	CGGGGATCCGTCGACCTG	
1629	CCCGTTTTTCTTTGCGACTAGACTAGCTATTTTTTGTATGGTTCATCCATGCCATG	
1634	GTTGGTGCGGATTCGTGATGC	

Table S6: Description of all set parameters and initial concentrations used for the modelling of microfluidic co-culture growth.

Parameter	Description	Value	Unit	Source
$\mu_{max-E.coli}$	Maximum growth rate of *E. coli* Wildtype in monoculture	0.25	1/h	This work
$\mu_{min-E.coli}$	Maximum growth rate of *E. coli* Δ*entC* in monoculture	0.15	1/h	This work
$\mu_{max-C.glutamicum}$	Maximum growth rate of *C. glutamicum* on glucose	0.4	1/h	(Unthan, Grünberger et al. 2014)
$k_{S,X2}$	kS value of enterobactin uptake by *E. coli*	0.2678	mg/L	Thulasiraman, Newton Salete et al. (1998)
$k_{S,X1}$	kS value of enterobactin uptake by *C. glutamicum*	10 x k_{S-E-E}	mg/L	Adjusted to check our hypothesis
$q_{max_{p-1-E.coli}}$	Production of abstract enterobactin-iron product at maximum growth rate of *E. coli*	90% of k_{S-E-C}	mg/L*h	Adjusted to check our hypothesis
$q_{max_{c-1-E.coli}}$	Consumption of abstract enterobactin-iron product at maximum growth rate of *E. coli*	0.4 x of $q_{max_{p-1-E.coli}}$	mg/L*h	Adjusted to check our hypothesis
$q_{max_{c-1-C.glut}}$	Consumption of abstract enterobactin-iron product at maximum growth rate of *C. glutamicum*	1.6 x of $q_{max_{c-1-E.coli}}$	mg/L*h	Adjusted to be the same differences as the growth rates of the organisms
$a_{\emptyset}$	Average cell area of a single cell	2.0	μm^2	This work
c_{cdw-c}	Average mass of cell dry weight by volume unit of cell	250	g/L	Smaluch, Wollenhaupt et al. (2023)
V_{M-P-C}	Volume of a microfluidic perfusion chamber	6000	μm^3	This work
d	Cell diameter and chamber height	1	µm	This work
P_0	Enterobactin concentration at the start	15	mg/L	Adjusted to check our hypothesis
$X_{Total-t_0}$	Total cell dry weight concentration at the start of the simulation	Varied	g/L	Varied to account for different start cell areas and compositions

Table S7: Nomenclature of modelled parameters.

Parameter	Descripton	Unit
$\mu_{max-E.coli}$	Maximum growth rate of *E. coli* Wildtype in monoculture	[1/h]
$\mu_{min-E.coli}$	Maximum growth rate of *E. coli* Δ*entC* in monoculture	[1/h]
A_{cell}	Average area of a single cell	[µm²]
$A_{cylinder}$	Projected area of the cylindrical part of the average cell	[µm²]
A_{sphere}	Projected area of the spherical part of the average cell	[µm²]
A_t	Observed total projected cell area at timepoint *t* for an entire chamber	[µm²]
$c_{A\rightarrow V}$	Factor to calculate cell area to cell volume. Describes how much cell volume is existing per µm² of projected cell area.	[µm³/µm²]
c_{cdw-c}	Average mass of cell dry weight by volume unit of cell	[g/L]
d	Cell diameter and chamber height	[µm]
k_{S-E-C}	kS value of enterobactin-iron product uptake by *C. glutamicum*	[mg/L]
k_{S-E-E}	kS value of enterobactin-iron product uptake by *E. coli*	[mg/L]
$L_{cylinder}$	Length of the cylindrical part of the average cell	[µm]
$P_{Enterobactin}$	Concentration of abstract enterobactin-iron product	[g/L]
P_0	Enterobactin-iron product concentration at the start	[mg/L]
$q_{max_{c-1-C.glut}}$	Consumption of abstract enterobactin-iron product at maximal growth rate of *C. glutamicum*	[mg/(L*h)]
$q_{max_{c-1-E.coli}}$	Consumption of abstract enterobactin-iron product at maximal growth rate of *E. coli*	[mg/(L*h)]
$q_{max_{p-1-E.coli}}$	Production of abstract enterobactin-iron product at maximal growth rate of *E. coli*	[mg/(L*h)]
V_{sphere}	Volume of the spherical part of the cell	[µm³]
$X_{C.glutamicum}$	Cell dry weight concentration of *C. glutamicum*	[g/L]
$X_{E.coli}$	Cell dry weight concentration of *E. coli*	[g/L]
X_{Total}	Total cell dry weight concentration, combination of both organisms	[g/L]
$X_{Total-t_0}$	Total cell dry weight concentration at the start of the simulation	[g/L]
X_t	Cell dry weight concentration at time point t	[g/L]

Formula S1: Derivation of conversion factor between measured cell area and volume of cells in the microfluidic system.

Three conversion steps were performed to convert observed cell areas into simulated cell dry weight concentrations. First the volume of all cells in the system is calculated by the measured cell area. Cells are assumed to be of the same average cell area and of the same geometry, which does not change over time. The cell morphology was described by a cylinder capped by two hemispheres. The diameter of the cell is assumed to be same as the height of the microfluidic system. A conversion factor is then used to calculate any measured projected cell area to cell volume.

$$A_{sphere} = \pi \times \frac{d}{2}^2 \tag{1}$$

$$A_{cylinder} = A_{cell} - A_{sphere} \tag{2}$$

$$L_{cylinder} = \frac{A_{cylinder}}{d} \tag{3}$$

$$V_{sphere} = \frac{4}{3} \times \pi \times \frac{d}{2}^3 \tag{4}$$

$$V_{cylinder} = \frac{d}{2}^2 \times \pi \times L_{cylinder} \tag{5}$$

$$V_{cell} = V_{sphere} + V_{cylinder} \tag{6}$$

$$c_{A \to V} = \frac{V_{cell}}{A_{cell}} \tag{7}$$

$$c_{A \to V} = \frac{\frac{4}{3} \times \pi \times \frac{d}{2}^3 + \frac{d}{2}^2 \times \pi \times \frac{A_{cell} - \pi \times \frac{d}{2}^2}{d}}{A_{cell}} \tag{8}$$

The calculated cell volume is then processed with an average value for cell dry weight per volume to cell dry weight. Lastly the cell dry weight concentration in a chamber is calculated by using the volume of a microfluidic chamber.

$$X_t = \frac{A_t \times c_{A \to V} \times c_{cdw-c}}{V_{M-P-C}} \tag{9}$$

$$X_{total} = X_{C.glut} + X_{E.coli} \tag{10}$$

Method: MS-measurements of enterobactin and organic acids

For extraction, 1 mL of solvent consisting of acetonitrile, methanol, and 0.1 M formic acid (40:40:20, v/v) was added to reaction tubes containing nylon filters with collected cells. The extraction solvent was supplemented with thio-alpha-ATP (TriLink Biotechnologies, 0.5 µM in 10 mL), d8- valine and d5-phenylalanine (Cambridge Isotope, each 0.5 µM in 10 mL extraction buffer), which served as internal standards. Cell disruption was achieved using a bead mill (Retsch) operated at 30 Hz for 2 minutes with pre-chilled holders to facilitate cell detachment from the filters and ensure efficient lysis. The resulting extract was neutralized by adding 90 µL of 15% (w/v) ammonium hydroxide.

After removal of the nylon filters and steel beads, samples were incubated at −20 °C for 30 minutes to improve protein precipitation. The samples were then centrifuged at 16,000 × g for 10 minutes at 4 °C, and the clarified supernatant was transferred to a 15 mL tube and diluted with 5 mL of ice-cold water. The diluted extracts were frozen at −80 °C and subsequently lyophilized for 48 hours using an Alpha 1–2 LDplus freeze dryer (CHRIST). Dried samples were stored at −80 °C and reconstituted in 200 µL of water prior to analysis.

For analysis of extracellular enterobactin, culture media was filtrated through a centrifugal filter with a size exclusion of 0.2 µm (VWR) and the cleared extract was immediately frozen in liquid nitrogen. For purification of enterobactin from the culture media, the filtrate was further extracted using solid-phase extraction (Strata-X polymeric reverse phase, Phenomenex) in accordance with the manufacturer's instructions. d8- valine and d5-phenylalanine (Cambridge Isotope) was added to the culture medium prior SPE as internal standard to a final concentration of 2.5 µM each. SPE eluate was transferred to a 15 mL tube and diluted with 5 mL of ice-cold water. The diluted eluates were frozen at −80 °C and subsequently lyophilized for 48 hours using an Alpha 1–2 LDplus freeze dryer (CHRIST). Dried samples were stored at −80 °C and reconstituted in 200 µL of water prior to analysis.

Enterobactin measurements were performed on an ACQUITY Premier reverse phase UPLC system (Waters) coupled to a Q Exactive Plus quadrupole–Orbitrap mass spectrometer (Thermo Scientific). An injection volume of 5 to 10 µL was introduced into the reverse phase UPLC system. Compounds were

separated on an ACQUITY UPLC HSS T3 analytical column (1.8 µm particle size VanGuard FIT, 2.1 mm x 150 mm, Waters) and achieved by generation of a solvent gradient (flow rate: 0.300 ml/min, 0-0.5 min 100% A, 0.5-6 min 100-89% A, 6-8 min 89-0% A, 8-11 min 0% A, 11-12 min 0-100%, 12-17 min 100% A). Solvent A consists of 10 mM Ammonium formiate in 0.15% formic acid and solvent B of 0.1% formic acid in acetonitrile. The column temperature was maintained at 35 °C. Ionization was performed in negative electrospray ionization (ESI) mode with the following source settings: sheath gas 30, auxiliary gas 15, sweep gas 2, spray voltage 3.2 kV, capillary temperature 230 °C, S-Lens RF level 45, and auxiliary gas heater temperature 280 °C. Full-scan mass spectra were acquired over an m/z range of 150–2000 at a resolution of 70,000. The automatic gain control target was set to 1×10^6 ions, with a maximum injection time of 200 ms. Targeted data processing was carried out using Skyline software (version 25.1, MacCoss Lab, University of Washington). Enterobactin was identified as a singly negatively charged species (m/z 668.1369) by high-resolution mass spectrometry, using a reference standard (Sigma-Aldrich, E3910). Enterobactin peak areas were normalized to the internal standards and to OD_{600} values recorded at the time of sampling.

Organic acids were analysed using a Dionex ICS-6000 HPIC system coupled to a Thermo Scientific Q Exactive Plus quadrupole–Orbitrap mass spectrometer, as described by (Schwaiger, Rampler et al. 2017). A 10 µL aliquot of the extracted sample was injected into the IC system using a Dionex AS-AP autosampler operated in push partial mode.

Anion exchange chromatography was performed using a Dionex IonPac AS11-HC analytical column (2 mm × 250 mm, 4 µm particle size; Thermo Fisher Scientific) equipped with a Dionex IonPac AG11-HC guard column (2 mm × 50 mm, 4 µm; Thermo Fisher Scientific) and operated at a column temperature of 30 °C. The mobile phase was generated using an eluent generator with a potassium hydroxide cartridge, producing a potassium hydroxide gradient. The flow rate was set to 380 µL min^{-1}, starting at a KOH concentration of 5 mM for 1 min, followed by a linear increase to 85 mM over 35 min, which was held for 5 min. The concentration was then immediately reduced to 5 mM, and the system was equilibrated for 10 min.

Spray stability of aqueous solution was improved using a makeup flow of methanol containing 10 mM acetic acid, delivered by an AXP pump with a flow rate of 150 µL min^{-1}. Electrospray ionisation was performed in the ESI source using the following parameters: sheath gas 30, auxiliary gas 15, sweep gas 0, spray voltage −2.8 kV, capillary temperature 300 °C, S-Lens RF level 45, and auxiliary gas heater temperature 380 °C.

Full-scan spectra were acquired over an m/z range of 60–800 at a resolution of 140,000, with an automatic gain control (AGC) target of 1×10^6 ions and a maximum injection time of 500 ms. Organic acids were identified according their retention time as a singly negatively charged species (fumaric acid m/z = 115.0037, citric and isocitric acid m/z 191.0197, succinic acid m/z = 117.0193, aconitic acid m/z = 173.0092, malic acid m/z = 133.0143, oxoglutaric acid m/z = 145.0143) by high-resolution mass spectrometry and using reference standards obtained from Sigma-Aldrich.

Targeted data evaluation was performed using Skyline (version 25.1, MacCos Lab Software, University of Washington). Peak areas were normalized to the internal standards and to OD_{600} values recorded at the time of sampling.

References

Baumgart, M., K. Luder, S. Grover, C. Gätgens, G. S. Besra and J. Frunzke (2013). "IpsA, a novel LacI-type regulator, is required for inositol-derived lipid formation in *Corynebacteria* and *Mycobacteria*." BMC Biol **11**: 122.

Boyer, H. W. and D. Roulland-Dussoix (1969). "A complementation analysis of the restriction and modification of DNA in *Escherichia coli*." J Mol Biol **41**(3): 459-472.

Cherepanov, P. P. and W. Wackernagel (1995). "Gene disruption in *Escherichia coli*: TcR and KmR cassettes with the option of Flp-catalyzed excision of the antibiotic-resistance determinant." Gene **158**(1): 9-14.

Choi, K.-H. and H. P. Schweizer (2006). "mini-Tn7 insertion in bacteria with single attTn7 sites: example Pseudomonas aeruginosa." Nature Protocols **1**(1): 153-161.

Choi, K. H., J. B. Gaynor, K. G. White, C. Lopez, C. M. Bosio, R. R. Karkhoff-Schweizer and H. P. Schweizer (2005). "A Tn7-based broad-range bacterial cloning and expression system." Nat Methods **2**(6): 443-448.

Cremer, J., L. Eggeling and H. Sahm (1990). "Cloning the *dapA dapB* cluster of the lysine-secreting bacterium *Corynebacterium glutamicum*." Molecular and General Genetics MGG **220**(3): 478-480.

Datsenko, K. A. and B. L. Wanner (2000). "One-step inactivation of chromosomal genes in *Escherichia coli* K-12 using PCR products." Proceedings of the National Academy of Sciences **97**(12): 6640-6645.

Figurski, D. H. and D. R. Helinski (1979). "Replication of an origin-containing derivative of plasmid RK2 dependent on a plasmid function provided in trans." Proc Natl Acad Sci U S A **76**(4): 1648-1652.

Kinoshita, S., S. Udaka and M. Shimono (2004). "Studies on the amino acid fermentation. Part 1. Production of L-glutamic acid by various microorganisms." J Gen Appl Microbiol **50**(6): 331-343.

Küberl, A., A. Mengus-Kaya, T. Polen and M. Bott (2020). "The Iron Deficiency Response of *Corynebacterium glutamicum* and a Link to Thiamine Biosynthesis." Appl Environ Microbiol **86**(10).

Mustafi, N., A. Grünberger, R. Mahr, S. Helfrich, K. Nöh, B. Blombach, D. Kohlheyer and J. Frunzke (2014). "Application of a Genetically Encoded Biosensor for Live Cell Imaging of L-Valine Production in Pyruvate Dehydrogenase Complex-Deficient *Corynebacterium glutamicum* Strains." PLOS ONE **9**(1): e85731.

Nelson, K. E., C. Weinel, I. T. Paulsen, R. J. Dodson, H. Hilbert, V. A. Martins dos Santos, D. E. Fouts, S. R. Gill, M. Pop, M. Holmes, L. Brinkac, M. Beanan, R. T. DeBoy, S. Daugherty, J. Kolonay, R. Madupu, W. Nelson, O. White, J. Peterson, H. Khouri, I. Hance, P. Chris Lee, E. Holtzapple, D. Scanlan, K. Tran, A. Moazzez, T. Utterback, M. Rizzo, K. Lee, D. Kosack, D. Moestl, H. Wedler, J. Lauber, D. Stjepandic, J. Hoheisel, M. Straetz, S. Heim, C. Kiewitz, J. A. Eisen, K. N. Timmis, A. Düsterhöft, B. Tümmler and C. M. Fraser (2002). "Complete genome sequence and comparative analysis of the metabolically versatile *Pseudomonas putida* KT2440." Environ Microbiol **4**(12): 799-808.

Panke, S., B. Witholt, A. Schmid and M. G. Wubbolts (1998). "Towards a biocatalyst for (S)-styrene oxide production: characterization of the styrene degradation pathway of *Pseudomonas sp. strain* VLB120." Appl Environ Microbiol **64**(6): 2032-2043.

Papadopoulos, A., M. T. Anlauf, J. Reiners, S.-H. Paik, A. Krüger, B. Lückel, M. Bott, T. Drepper, J. Frunzke, H. Gohlke, S. Weidtkamp-Peters, S. H. J. Smits and C. G. W. Gertzen (2026). "A Novel Biosensor for Ferrous Iron Developed via CoBiSe: A Computational Method for Rapid Biosensor Design." ACS Sensors **11**(1): 119-135.

Schäfer, A., A. Tauch, W. Jäger, J. Kalinowski, G. Thierbach and A. Pühler (1994). "Small mobilizable multi-purpose cloning vectors derived from the *Escherichia coli* plasmids pK18 and pK19: selection of defined deletions in the chromosome of *Corynebacterium glutamicum*." Gene **145**(1): 69-73.

Schwaiger, M., E. Rampler, G. Hermann, W. Miklos, W. Berger and G. Koellensperger (2017). "Anion-Exchange Chromatography Coupled to High-Resolution Mass Spectrometry: A Powerful Tool for Merging Targeted and Non-targeted Metabolomics." Analytical Chemistry **89**(14): 7667-7674.

Smaluch, K., B. Wollenhaupt, H. Steinhoff, D. Kohlheyer, A. Grünberger and C. Dusny (2023). "Assessing the growth kinetics and stoichiometry of Escherichia coli at the single-cell level." Engineering in Life Sciences **23**(1): e2100157.

Thulasiraman, P., M. C. Newton Salete, J. Xu, N. Raymond Kenneth, C. Mai, A. Hall, A. Montague Marjorie and E. Klebba Phillip (1998). "Selectivity of Ferric Enterobactin Binding and Cooperativity of Transport in Gram-Negative Bacteria." Journal of Bacteriology **180**(24): 6689-6696.

Unthan, S., A. Grünberger, J. van Ooyen, J. Gätgens, J. Heinrich, N. Paczia, W. Wiechert, D. Kohlheyer and S. Noack (2014). "Beyond growth rate 0.6: What drives *Corynebacterium glutamicum* to higher growth rates in defined medium." Biotechnology and Bioengineering **111**(2): 359-371.
